# Modeling lacto-vegetarian, pescatarian, and “pescavegan” USDA food patterns and assessing nutrient adequacy for healthy non-pregnant, non-lactating adults

**DOI:** 10.3389/fnut.2023.1113792

**Published:** 2023-02-07

**Authors:** Julie M. Hess, Madeline E. Comeau

**Affiliations:** USDA-ARS Grand Forks Human Nutrition Research Center, Grand Forks, ND, United States

**Keywords:** diet, nutrients, nutrition guidelines, vegetarian, vegan, pescatarian

## Abstract

**Introduction:**

The 2020–2025 Dietary Guidelines for Americans (DGA) includes a Healthy Vegetarian Dietary Pattern (HVDP) with dairy foods and eggs as one of its three recommended dietary patterns for non-pregnant, non-lactating healthy adults. This study evaluates whether pescatarian, lacto-vegetarian, and “pescavegan” adaptations of the HVDP can be nutritionally adequate if modeled with foods recommended by the DGA.

**Methods:**

The nutrient composition of these three alternative models of the HVDP were assessed at 1, 800-, 2, 000-, 2, 200-, and 2,400- kcal/day using similar food pattern modeling procedures as the 2020 DGA. For the pescatarian and pescavegan models, 0.5 ounce-equivalent of refined grains per day was replaced with seafood. For the lacto-vegetarian and pescavegan models, eggs were replaced with equal proportions of the other vegetarian protein foods. In the pescavegan model, dairy foods were replaced by a dairy alternative group comprised of fortified soy milk and soy yogurt.

**Results:**

All models at all energy levels were within Acceptable Macronutrient Distribution Ranges (AMDRs) for all macronutrients, contained ≤5% of total kcal from saturated fat, and met recommendations for most micronutrients. Nutrients provided below the Dietary Reference Intakes (DRIs) in these models included iron, sodium, vitamin D, vitamin E, and choline. Micronutrients provided at less than 50% of their respective DRIs included vitamin D and choline.

**Discussion:**

Adapting the HVDP for lactovegetarian, pescatarian, and pescavegan dietary patterns provided adequate amounts of macronutrients and most micronutrients.

## Introduction

The 2020–2025 Dietary Guidelines for Americans (DGA) recommends three healthy dietary patterns- a Healthy U.S.-Style Dietary Pattern (HUSDP), a Healthy Mediterranean-Style Dietary Pattern, and a Healthy Vegetarian Dietary Pattern (HVDP) (1). In all of these patterns, the “majority of energy (comes from) plant-based foods, such as vegetables, fruits, legumes, whole grains, nuts, and seeds” (2). These “plant-based” DGA patterns promote health and reduce the risk of chronic disease across the lifespan, including a lower risk of cardiovascular disease, total and LDL cholesterol, blood pressure, and type 2 diabetes (1). While previous cross-sectional studies of the American population indicate that most Americans follow omnivorous diets including self-described vegetarians and vegans (2), data from the National Health and Nutrition Examination Survey (NHANES) on how Americans identify their dietary patterns has not been updated for over a decade (3, 4). Meanwhile, interest in and research about different iterations of vegetarian dietary patterns has increased (5). A study that used NHANES data from 2013 to 2016 found that approximately 7% of adults ate a diet without meat, poultry, or fish on day 1 of their dietary recall (6).

Regardless of how many Americans follow vegetarian eating patterns, the DGA are intended to provide dietary advice for all healthy Americans, which includes those who choose to follow different iterations of vegetarian dietary patterns. The HVDP is a lacto-ovo vegetarian pattern that includes eggs and dairy foods. However, there are vegetarians who avoid eggs, vegetarians who eat fish (pescatarians), and vegetarians who avoid all animal products except fish (pescavegans or “seagans”). Table 1 lists the dietary restrictions and preferences of different types of vegetarian eating patterns. Vegetarians and vegans may be at risk for underconsuming certain nutrients that are more readily available in animal-based foods. For instance, Americans who follow vegetarian dietary patterns may be at risk of underconsuming iron, vitamin B12, vitamin D, omega-3 fatty acids, zinc, calcium, and iodine (5, 7, 8).

**TABLE 1:** Eating pattern descriptions.

Type of diet	Restrictions
Omnivorous	None
Pescatarian	Avoid meat and poultry
Vegetarian or lacto-ovo-vegetarian	Avoid meat, poultry, seafood
Ovo-vegetarian	Avoid meat, poultry, seafood, dairy
Lacto-vegetarian	Avoid meat, poultry, seafood, eggs
Pescavegan	Avoid meat, poultry, eggs, dairy
Vegan	Avoid all animal products

While little is known about the relative populations of these groups, the benefits of seafood have often been discussed in the scientific literature. Depending on the reason for adopting a vegan diet, some vegans still choose to eat some or all kinds of fish or seafood. For instance, ostrovegans and pescavegans continue to consume oysters or all seafood, respectively, in addition to a traditional vegan diet (9). Neither term is well-known or widely adopted (10–12). Yet, “oysters are considered (almost) fair game by plenty of animal ethicists,” some of whom are vegan, as potentially “morally preferable” to certain plant foods (13). There is no data at this time for how many Americans may be eating vegan diets that include some seafood.

The benefits of regular seafood consumption are discussed in some depth in evidence-based nutrition guidance from both the 2015 and 2020 Dietary Guidelines Scientific Advisory Committee Reports (DGAC) and the American Heart Association (AHA) Scientific Advisory Group (2, 14, 15). The 2015 DGAC reported that strong evidence indicated that seafood intake was associated with a reduced risk of cardiovascular disease (14), and the 2020 DGAC continued to recommend that Americans 2 years and older consume two or more cooked servings of seafood per week to “ensure intake of key nutrients and as part of an overall healthy dietary pattern” (2). The AHA Scientific Advisory Group also states that, among other associations with fish intake and various cardiometabolic parameters, “consuming ∼1 to 2 fatty fish meals per week is associated with a 50% or lower risk of sudden cardiac death compared with little or no seafood intake after adjustment for potentially confounding factors” (15).

Yet, seafood is a relatively small part of the U.S. diet, when viewed on an average per capita consumption level (2). Among children 2 to 19 years, seafood comprises approximately 0.2 ounce-equivalents (oz-eq) per day of protein food intake (2), and, among adults 19 and older, seafood contributes only 0.6 oz-eq per day. The DGA, however, encourages intake of 8 oz-eq of seafood per week for people eating a 2,000 kcal HUSDP. In particular, the 2020 DGAC notes that seafood, which includes fish (e.g., salmon, tuna, trout, tilapia) and shellfish (e.g., shrimp, crabs, oysters), is a key contributor to omega-3 fatty acid intake (2). However, supplementing a vegetarian or vegan diet- including the HVDP from the 2020 DGA- with seafood has not been modeled from a nutrient adequacy perspective, which is the aim of this study.

## Materials and methods

### Lacto-vegetarian dietary pattern (model 1)

To adapt the HVDP from the 2020 DGA for a lacto-vegetarian diet, eggs were replaced in the 1,800, 2,000, 2,200, and 2,400 kcal HVDP with equivalent amounts of the other vegetarian protein sources- nuts and seeds, soy products, and beans, peas, and lentils.

### Pescatarian dietary pattern (model 2)

To adapt the HVDP from the 2020 DGA for a pescatarian diet, we added the recommended amounts of seafood from the HUSDP to the HVDP at the 1,800, 2,000, 2,200, and 2,400 kcal levels, at which seafood recommendations range from 8 to 10 oz-eq per week.

The Food Pattern Modeling (FPM) report from the 2020 DGAC (16) includes nutrient profiles of a low omega-3 fish group and a high omega-3 fish group. However, the HUSDP and the Healthy Mediterranean-Style Dietary Pattern in the 2020 DGA (1) group these two types of fish together as a “seafood group.” Because the process of combining the low- and high-omega-3 fish nutrient profiles into a single food group is not detailed in the FPM report, for this study, we combined these two “fish” groups into a single “seafood” group based on weighted consumption patterns (e.g., high omega-3 fish comprise roughly 31% of the seafood consumed by Americans and low omega-3 fish comprise roughly 69% of the seafood consumed by Americans). We then used this “seafood group” in the food pattern modeling for our pescatarian and pescavegan dietary pattern adaptations. The seafood group nutrient profile can be found in Supplementary Table 1.

The addition of 9 oz-eq servings of seafood per week in the 2,000 kcal model adds approximately 50 calories to the diet per day. To mitigate the increase in calories, we decreased the servings of refined grains by 0.5 oz-eq daily (from 3.5 oz-eq per day to 3 oz-eq per day in the 2000 kcal model), a decrease of approximately 43 calories. This change (decrease in refined grain servings by 0.5 oz-eq) was made across all calorie levels in models containing seafood (pescatarian and pescavegan models).

### Pescavegan dietary pattern (model 3)

For the pescavegan pattern, we used a similar process of adding 8 to 10 oz-eq of seafood per week to the HVDP at the 1,800, 2,000, 2,200, and 2,400 kcal levels and decreasing the daily amount of refined grain foods by 0.5 oz-eq. In addition to this change, we also replaced the amounts of dairy foods with the dairyALT group, comprised of fortified soy milk and soy yogurt, as described in a previous publication (5) (Supplementary Table 2) and replaced eggs with equivalent amounts of the other vegetarian protein sources including nuts and seeds, soy products, and beans, peas, and lentils. An overview of the HVDP adaptations in this manuscript can be found in Table 2.

**TABLE 2:** From the 2020 Dietary Guidelines for Americans: daily or weekly amounts for each food group in the 2,000-kcal/d Healthy Vegetarian Dietary Pattern compared with the modeled dietary patterns^1^.

Food groups	HVDP	Model 1: lacto-vegetarian	Model 2: pescatarian	Model 3: pesca-vegan
Vegetables, cup eq/d	2.5	2.5	2.5	2.5
Dark green, cup eq/wk	1.5	1.5	1.5	1.5
Red and orange, cup eq/wk	5.5	5.5	5.5	5.5
Beans, peas, lentils, cup eq/wk	1.5	1.5	1.5	1.5
Starchy vegetables, cup eq/wk	5.0	5.0	5.0	5.0
Other vegetables, cup eq/wk	4.0	4.0	4.0	4.0
Fruits, cup eq/d	2.0	2.0	2.0	2.0
Grains, oz eq/d	6.5	6.5	6.5	6.5
Whole grains	3.5	6.5	3.5	3.5
Refined grains	3.0	3.0	2.5	2.5
Dairy, cup eq/d	3.0	3.0	3.0	0.0
DairyALT, cup eq/d	0.0	0.0	0.0	3.0
Protein foods, oz eq/d	3.5	3.5	4.64	4.64
Eggs, oz eq/wk	3.0	0.0	3.0	0.0
Beans, peas, lentils, oz eq/wk	6.0	7.0	6.0	7.0
Soy products, oz eq/wk	8.0	9.0	8.0	9.0
Nuts, seeds, oz eq/wk	7.0	8.0	7.0	8.0
Seafood (oz eq/day)	0.0	0.0	8.0	8.0
Oils, g/day	27	27	27	27
Discretionary calories (kcal/d)	250	250	250	250

^1^One cup eq=2.37 dL. 1 oz eq=28.35 g. Cup eq, cup equivalent; DairyALT, dairy alternative; HVDP, Healthy Vegetarian Dietary Pattern; oz eq, ounce equivalent.

## Results

All models at all energy levels were within Acceptable Macronutrient Distribution Ranges (AMDRs) for all macronutrients (Table 3), contained ≤5% of total kcal from saturated fat, and met most micronutrient requirements for the Dietary Reference Intakes (DRI) for the population groups for whom the different energy levels may be appropriate. For instance, the three models at the 1,800 kcal level were compared to DRIs for females 31–50 y, and the 2,000 kcal models were compared to DRIs for females 19–30 y and males 51 + y, because the 2020 DGA specifies that 1,800 and 2,000 kcal dietary patterns may be appropriate for these population groups (1). Similarly, the 2,200 kcal models were compared to DRIs for males 31–50 y, and the 2,400 kcal models were compared to DRIs for males 19–30 y (1). Amounts of calcium, phosphorus, copper, selenium, vitamin C, thiamin, riboflavin, niacin, vitamin B6, vitamin B12, and folate met DRIs across all energy levels of all three models.

**TABLE 3:** Acceptable Macronutrient Distribution Ranges in percentage of total calories.

**1,800 Healthy Vegetarian Dietary Pattern (total kcal 1,797)**	
Protein	16.9%
Carbohydrate	52.8%
Fat	25.0%
**1,800 model 1: lacto-vegetarian model (total kcal 1,790)**	
Protein	16.8%
Carbohydrate	53.2%
Fat	24.6%
**1,800 model 2: pescatarian model (total kcal 1,799)**	
Protein	18.2%
Carbohydrate	50.9%
Fat	25.5%
**1,800 model 3: pescavegan model (total kcal 1,866)**	
Protein	15.6%
Carbohydrate	50.2%
Fat	28.0%
**2,000 Healthy Vegetarian Dietary Pattern (total kcal 1,998)**	
Protein	16.0%
Carbohydrate	50.0%
Fat	24.4%
**2,000 model 1: lacto-vegetarian model (total kcal 1,991)**	
Protein	16.1%
Carbohydrate	50.4%
Fat	24.0%
**2,000 model 2: pescatarian model (total kcal 2,000)**	
Protein	16.0%
Carbohydrate	50.0%
Fat	25.2%
**2,000 model 3: pescavegan model (total kcal 2,067)**	
Protein	15.1%
Carbohydrate	47.8%
Fat	27.0%
**2,200 Healthy Vegetarian Dietary Pattern (total kcal 2,201)**	
Protein	15.4%
Carbohydrate	50.3%
Fat	23.9%
**2,200 model 1: lacto-vegetarian model (total kcal 2,197)**	
Protein	15.5%
Carbohydrate	50.7%
Fat	23.8%
**2,200 model 2: pescetarian model (total kcal 2,209)**	
Protein	16.6%
Carbohydrate	48.7%
Fat	24.4%
**2,200 model 3: pescavegan model (total kcal 2,279)**	
Protein	14.7%
Carbohydrate	48.3%
Fat	26.4%
**2,400 Healthy Vegetarian Dietary Pattern (total kcal 2,404)**	
Protein	15.1%
Carbohydrate	49.4%
Fat	23.7%
**2,400 model 1: lacto-vegetarian model (total kcal 2,400)**	
Protein	15.2%
Carbohydrate	49.8%
Fat	23.2%
**2,400 model 2: pescatarian model (total kcal 2,422)**	
Protein	16.4%
Carbohydrate	47.7%
Fat	24.4%
**2,400 model 3: pescavegan model (total kcal 2,488)**	
Protein	14.6%
Carbohydrate	47.4%
Fat	26.0%

Adults ages 19 +: Protein = 10–35% of calories, Carbohydrate = 45–65% of calories, Fat = 20–35% of calories.

Tables 4–6 as well as Supplementary Tables 3–14 show the results of these adaptations (Models 1–3) compared to both the original HVDP as well as to the DRIs for different population groups (1). These tables show percentage changes in nutrient content between the HVDP and the lacto-vegetarian, pescatarian, and pescavegan modeled alternatives as well as the percentage of the DRIs met by these HVDP adaptations.

**TABLE 4:** Impact of replacing eggs in the 2,000 kcal/d Healthy Vegetarian Dietary Pattern (HVDP) with vegetarian protein group foods on Dietary Reference Intakes (DRIs) for females 19–30 y.

Nutrients	DRIs: females 19–30	HVDP	Model 1: lacto-vegetarian	Change from HVDP,%	Percentage (%) of DRI met by model 1
**Macronutrients**					
Calories kcal	2,000	1,998	1,991	-0.34	99.55
Protein (g)	56	80	80	0.21	142.86
Carbohydrate (g)	130	250	251	0.48	193.08
Fiber (g)	28	30	31	3.55	110.71
Total fat (g)	20–35%	54	53	-2.46	Within range
Saturated fat (g)	<10%	10	10	-2.30	Within limits
Monounsaturated fat (g)	n/a	20	19	-2.60	n/a
Polyunsaturated fat (g)	n/a	21	21	1.82	n/a
Linoleic acid (g)	17	18	18	-1.02	105.88
Linolenic acid (g)	1.6	2.3	2.4	2.47	218.18
EPA (g)	n/a	0.000	0.000	n/a	n/a
DHA (g)	n/a	0.009	0.000	n/a	n/a
Cholesterol (mg)	as low as possible	105	25	-76.24	n/a
**Minerals**					
Calcium (mg)	1,000	1,341	1,342	0.09	134.20
Iron (mg)	18	16	17	3.49	94.44
Magnesium (mg)	310	381	387	1.45	124.84
Phosphorus (mg)	700	1,609	1,604	-0.29	229.14
Potassium (mg)	2,600	3,272	3,282	0.31	126.23
Sodium (mg)	2,300	1,461	1,455	-0.43	63.26
Zinc (mg)	8	11	11	-3.98	137.50
Copper (mg)	0.9	1.6	1.7	4.12	188.89
Selenium (mcg)	55	79	73	-7.81	132.73
**Vitamins**					
Vitamin A, RAE (mcg)	700	847	815	-3.75	116.43
Vitamin E, AT (mg)	15	10	10	-2.36	66.67
Vitamin D (IU)	600	220	202	-8.08	33.67
Vitamin C (mg)	75	129	130	0.40	173.33
Thiamin (mg)	1.1	1.8	1.8	0.64	163.64
Riboflavin (mg)	1.1	1.8	1.8	-1.28	164.64
Niacin (mg)	14	17	17	3.01	121.43
Vitamin B6 (mg)	1.3	1.8	1.8	-1.76	138.46
Vitamin B12 (mcg)	2.4	3.9	3.6	-6.51	150.00
Choline (mg)	425	300	243	-18.88	57.18
Vitamin K (mcg)	90	139	139	0.36	154.44
Folate, DFE (mcg)	400	612	616	0.65	154.00

**TABLE 5:** 2,000 kcal pescatarian model of the Healthy Vegetarian Dietary Pattern (HVDP) compared to Dietary Reference Intakes (DRIs) for females 19–30 y.

Nutrients	DRIs: females 19–30	HVDP	Model 2: pescatarian	Change from HVDP,%	Percentage (%) of DRI met by model 2
**Macronutrients**					
Calories kcal	2,000	1,998	2,000	0.11	100.00
Protein (g)	56	80	87	8.97	155.36
Carbohydrate (g)	130	250	250	0.08	192.31
Fiber (g)	28	30	30	0.21	107.14
Total fat (g)	20–35%	54	56	3.06	within range
Saturated fat (g)	<10%	10	11	7.46	within limit
Monounsaturated fat (g)	n/a	20	20	2.53	n/a
Polyunsaturated fat (g)	n/a	21	21	1.82	n/a
Linoleic acid (g)	17	18	18	-1.02	105.88
Linolenic acid (g)	1.1	2.3	2.3	-1.80	209.09
EPA (g)	n/a	0.000	0.073	0.00	n/a
DHA (g)	n/a	0.009	0.165	1818.60	n/a
Cholesterol (mg)	as low as possible	105	131	24.48	n/a
**Minerals**					
Calcium	1,000	1,341	1,351	0.76	135.10
Iron (mg)	18	16	17	3.49	94.44
Magnesium (mg)	310	381	392	2.77	126.45
Phosphorus (mg)	700	1,609	1,693	5.24	241.86
Potassium (mg)	2,600	3,272	3,376	3.19	129.85
Sodium (mg)	2,300	1,461	1,546	5.80	67.22
Zinc (mg)	8	11	12	4.75	150.00
Copper (mg)	0.9	1.6	1.7	4.12	188.89
Selenium (mcg)	55	79	95	19.97	172.73
**Vitamins**					
Vitamin A, RAE (mcg)	700	847	859	1.44	122.71
Vitamin E, AT (mg)	15	10	11	7.41	73.33
Vitamin D (IU)	600	220	299	36.06	49.83
Vitamin C (mg)	75	129	130	0.40	173.33
Thiamin (mg)	1.1	1.8	1.7	-4.95	154.55
Riboflavin (mg)	1.1	1.8	1.8	-1.28	163.64
Niacin (mg)	14	17	18	9.07	128.57
Vitamin B6 (mg)	1.3	1.3	1.9	3.70	146.15
Vitamin B12 (mcg)	2.4	3.9	5.0	29.84	208.33
Choline (mg)	425	300	322	7.50	75.76
Vitamin K (mcg)	90	139	139	0.36	154.44
Folate, DFE (mcg)	400	612	618	0.98	154.50

**TABLE 6:** Impact of adding fish to the 2,000 kcal/d vegan adaptation of the Healthy Vegetarian Dietary Pattern (HVDP) on Dietary Reference Intakes (DRIs) for females 19–30 y.

Nutrients	DRIs: females 19–30 years	HVDP, 2,000 kcal	Model 3: pescavegan	Change from HVDP,%	Percentage (%) of DRI met by model 3
**Macronutrients**					
Calories kcal	2,000	1,998	2,067	3.47	103.35
Protein (g)	56	80	78	-2.30	139.29
Carbohydrate (g)	130	250	247	-1.12	190.00
Fiber (g)	28	30	31	3.55	110.71
Total fat (g)	20–35%	54	62	14.10	Within range
Saturated fat (g)	<10%	10	10	-2.30	Within limits
Monounsaturated fat (g)	n/a	20	21	7.65	n/a
Polyunsaturated fat (g)	n/a	21	27	30.91	n/a
Linoleic acid (g)	17	18	22	20.97	129.41
Linolenic acid (g)	1.6	2.3	2.8	19.55	254.55
EPA (g)	n/a	0.000	0.073	0.00	n/a
DHA (g)	n/a	0.009	0.156	1713.95	n/a
Cholesterol (mg)	As low as possible	105	29	-72.44	n/a
**Minerals**					
Calcium	1,000	1,341	1,325	-1.17	132.50
Iron (mg)	18	16	19	15.67	105.56
Magnesium (mg)	310	381	420	10.11	135.48
Phosphorus (mg)	700	1609	1296	-19.44	185.14
Potassium (mg)	2,600	3,272	3,422	4.59	131.62
Sodium (mg)	2,300	1,461	1,231	-15.76	53.52
Zinc (mg)	8	11	10	-12.71	125.00
Copper (mg)	0.9	2	2.7	65.37	300.00
Selenium (mcg)	55	79	80	1.03	145.45
**Vitamins**					
Vitamin A, RAE (mcg)	700	847	883	4.28	126.14
Vitamin E, AT (mg)	15	10.	11	7.41	73.33
Vitamin D (IU)	600	220	430	95.68	71.67
Vitamin C (mg)	75	129	137	5.81	182.37
Thiamin (mg)	1.1	1.8	1.7	-4.95	154.55
Riboflavin (mg)	1.1	1.8	2.0	9.69	181.82
Niacin (mg)	14	17	20	21.18	142.86
Vitamin B6 (mg)	1.3	1.8	1.9	3.70	146.15
Vitamin B12 (mcg)	2.4	3.9	7.7	99.96	320.83
Choline (mg)	425	300	345	15.17	81.18
Vitamin K (mcg)	90	139	158	14.08	175.56
Folate, DFE (mcg)	400	612	631	3.10	157.75

### 1,800 kcal models–Females 31–50 y

The pescatarian and lacto-vegetarian models provided 1,800 ± 10 kcal, and the pescavegan model provided 66 additional kcal (1,866 kcal). Vitamin D was below 50% of the DRI in the pescatarian model (49.63%) as well as the lacto-vegetarian model (33.50%). In the pescavegan model, 71.60% of the DRI for Vitamin D was provided. Other nutrients below the DRIs included iron in the pescatarian and lacto-vegetarian models and sodium, vitamin E, choline in all three models. These nutrients were provided in amounts ranging from 53 to 87% of the DRIs (Supplementary Tables 3–5).

### 2,000 kcal models–Females 19–30 y, males 51 + y

These models provided 2,000 ± 9 kcal, except the pescavegan model, which provided 67 additional kcal (2,067 kcal). For both males 51 + y and females 19–30 y, vitamin D was provided at less than 50% of the DRI in the pescatarian and lacto-vegetarian models. Vitamin D was provided at less than 100% of the DRI in the pescavegan models for both males and females (71.67%).

Iron in the pescatarian and lacto-vegetarian models and sodium, vitamin E, and choline across all three models were provided in amounts ranging from 53.52 to 94.44% of the DRIs for females 19–30 y.

For males 51 + y in the 2,000 kcal model, choline was provided at less than 50% of the DRI in the lacto-vegetarian model (44.18%). Other nutrients provided at levels below 100% of the DRIs (53.52–99.29%) included magnesium, potassium, sodium, vitamin A, vitamin E, and choline in the lacto-vegetarian and pescatarian models. The pescavegan model provided adequate nutrients with the exception of sodium (53.52% of the DRI), zinc (90.91%), vitamin A (98.11%), vitamin E (73.33%), vitamin D (71.67%), and choline (62.73%) (Tables 4–6 and Supplementary Tables 6–8).

### 2,200 kcal models–Males 31–50 y

These models provided 2,200 ± 9 kcal, except the pescavegan model, which provided 79 additional kcal (2,279 kcal). Vitamin D and choline were provided at levels below 50% of the DRI in the lacto-vegetarian model (34.17 and 47.64%, respectively).

Nutrients provided at less than 100% of the DRIs across all three models included sodium, vitamin E, vitamin D, and choline. These nutrients were provided in amounts ranging from approximately 52–99% of the DRIs (Supplementary Tables 9–11).

### 2,400 kcal models–Males 19–30 y

These models provided 2,400 ± 22 kcal, except the pescavegan model, which provided 88 additional kcal (2,488 kcal). Vitamin D was the only micronutrient below 50% of the DRIs in the lacto-vegetarian model only (34.67%). Vitamin D amounts were below100% of the DRI in the pescatarian (54.17%), and pescavegan (76.17%) models. Other nutrients provided at less than 100% of the DRIs included choline, vitamin E, and sodium (Supplementary Tables 12–14).

## Discussion

To our knowledge, this is the first study to model and evaluate the nutritional adequacy of pescatarian, lacto-vegetarian, or pescavegan adaptations to USDA Food Patterns recommended in the DGA. The pescatarian, lacto-vegetarian, and pescavegan models provided adequate amounts of vitamin B12 and calcium, which are often considered nutrients of concern in vegetarian diets. However, these models did not provide recommended amounts of other nutrients of concern for vegetarians, including vitamin D, iron, zinc, and choline (5, 7, 8). None of the dietary patterns in the DGA, including the HVDP, meet recommendations for choline and vitamin D. With regard to these nutrients, the adaptations created in this study are aligned with current DGA recommendations. Each of these nutrients is discussed in detail below as well as other potential nutrients to consider in vegetarian diets- omega-3 fatty acids, sodium, and iodine.

### Vitamin D

Across all calorie levels, the lacto-vegetarian model provided vitamin D in amounts less than 50% of the DRIs. The pescatarian and pescavegan models, however, provided between 50 and 99% of the DRIs for vitamin D for healthy adults in the age ranges for which our modeled dietary patterns may be appropriate. Both the pescatarian and pescavegan models contain fish, which can be a dietary source of vitamin D. Fish such as trout, salmon, and sardines are known sources of vitamin D (17). One oz-eq of the seafood group provided 69.82 IU of vitamin D that was not provided in the lacto-vegetarian models.

The pescavegan models provided the highest amount of vitamin D across all calorie levels due to the nutrient makeup of the dairyALT group. The dairyALT group contains just soy milk and soy yogurt, while the dairy group includes milk (49.6%), yogurt (4.5%), cheese (44.8%), and soy milk (1%). Cheese, comprising nearly half the dairy group, is not a source of vitamin D. Therefore, the dairyALT group contains over double the amount of vitamin D as the dairy group. It is worth noting that current FDA regulations (as of 2016), allow up to 84 IU/100 g of vitamin D2 to be added to plant-based beverages intended as milk alternatives, and 89 IU/100 g of vitamin D2 can be added to plant-based yogurt alternatives (18). Up to 84 IU/100 g of vitamin D3 can be added to dairy milk but not cheese and yogurt (18). For comparison, the vitamin D provided from 3 c-eq from the dairy group is 175.5 IU, where the same 3 c-eq of dairyALT provides 326.4 IU.

### Iron

Iron levels were adequate across all dietary patterns and all calorie levels appropriate for adult males. The DRI for males 51 + y (2,000 kcal model), 31–50 y (2,200 kcal model), and 19–30 y (2.400 kcal model) is 8 mg of iron per day. For females 31–50 y (1,800 kcal model) and 19–30 y (2,000 kcal model), the DRI for iron is 18 mg per day, higher than the adult male requirements. In both the 1,800 kcal/day (30–50 y females) and 2,000 kcal/day (19–30 y females) models, iron levels across the lacto-vegetarian and pescatarian patterns fell short. However, the pescavegan model provided adequate amounts of iron for females due to the iron content of the dairyALT group. For context, the original HVDP model provided adequate iron across all models for males and provided 86.74–91.26% of the DRI in the models for females. The dairyALT group provides 0.94 mg iron per one cup-equivalent, while the dairy group provides 0.08 mg/cup-eq. Our lacto-vegetarian models provided 16 mg (87.78%) of iron at the 1,800 kcal model and 17 mg (94.44%) at the 2,000 kcal model, and pescatarian models provided 15 mg (85.56%) and 17 mg iron (94.44%), respectively, at the 1,800 and 2,000 kcal levels. The pescavegan pattern, containing the dairyALT group, at both the 1,800 and 2,000 kcal levels provided at least 18 mg DRI, an adequate amount for adult females in these age categories. The nutrient profile of the dairyALT group provided enough iron in the pescavegan pattern to meet recommendations for pre-menopausal females, while the original dairy group does not. This difference indicates the importance of fortified soy products (milk and yogurt) as nutrient-dense alternatives to milk, cheese, and yogurt.

### Zinc

As with the vegan models in our previous study (5), the pescavegan model provided slightly less zinc than recommended for males at the 2,000 kcal level. This result may be due to few zinc-rich foods being plant-based. The Office of Dietary Supplements lists vegetarians, especially vegans, as a group most likely to have inadequate zinc status (19). Pescavegans, however, have more animal-based options than vegans, because they eat seafood. Some seafood choices, especially oysters, are good or excellent dietary sources of zinc. While the models in this manuscript were based on average American eating patterns, pescavegans could adapt their seafood choices to accommodate more zinc-rich options.

### Choline

Choline, another potential nutrient of concern in vegetarian diets, was lowest in the lacto-vegetarian patterns, in a range of 44.18–57.18% of the DRIs across calorie levels. The lacto-vegetarian model contains dairy as opposed to the dairyALT group. One cup-eq of dairy milk contains 25.67 mg choline compared to 52.89 mg in one cup-eq of the dairyALT group, nearly double what the dairy group provides. The reason for differing choline amounts between the pescatarian and lacto-vegetarian models, both of which include the dairy versus dairyALT group, is likely due to the pescatarian model containing seafood. In the 1.43 oz-eq of seafood/d in the 2,400 kcal pescatarian model, an additional 27.9 mg of choline is provided that is not present in the lacto-vegetarian model at the same calorie level. This difference can be observed across all calorie levels. Consistent with these findings, previous research has noted that it can be difficult to achieve adequate intakes of choline without eggs (20). Our sole model containing eggs, the pescatarian model, attained a range of 62.00–72.16% of the DRI for choline across calorie levels, which was greater than the choline amounts provided by the lacto-vegetarian models but similar to the choline amounts in the pescavegan models (62.73–81.18%).

### Omega-3 fatty acids

All three models across each calorie level provided some omega-3 fatty acids including alpha-linolenic acid (ALA), eicosapentaenoic acid (EPA), and docosahexaenoic acid (DHA). Currently, there is only an adequate intake (AI) value established for ALA due to insufficient data available to establish an estimated average requirement (EAR) for other omega-3 fatty acids. The AI for ALA is 1.1 g/day in females 14 + y, and 1.6 g/day in males 14 + y (21). Sufficient amounts of ALA were provided across all of our models, including both of the models containing fish as well as the lacto-vegetarian model throughout all calorie levels. In addition to fish, ALA is commonly found in plant foods such as flaxseed oil, chia seeds, walnuts, canola oil, and beans (21). The fish-containing models across all calorie levels also contained small amounts of both EPA and DHA, while the lacto-vegetarian model did not contain either of these omega-3 fatty acids, similar to the original HVDP models. Currently, the AHA recommends 2, 3-oz servings of fish per week to obtain adequate dietary omega-3 fatty acids (15).

### Sodium

Sodium levels were low across most of our models when compared to the DRI of 2,300 mg/day (51.73–75.57%). However, intakes of sodium for Americans generally range from 3,001 to 4,100 mg of sodium daily, with the average intake of around 3,393 mg per day (2). Our modeled iterations of the HVDP are likely not representative of the average sodium intake of Americans. The currently established Adequate Intake (AI) value for sodium is 1,500 mg/d for healthy adults, and the Chronic Disease Risk Reduction Intake (CDRR) for sodium is set at 2,300 mg/d. This CDRR value is intended as a guide for reducing sodium intake (22). While several of our models did not contain sodium amounts reaching the CDRR, most of our models did meet the AI.

### Iodine

Iodine is a commonly cited nutrient of concern for vegans and for those who eat few or no dairy products, seafood, or eggs and, therefore, these individuals are considered a subpopulation at risk for an iodine deficiency (23). Foods of marine origin tend to have higher concentrations of iodine, because these foods and/or animals concentrate iodine from seawater (24). While iodine is also a nutrient of concern in vegetarian diets, there is not currently data on iodine amounts in many foods, including the ones used to develop the FPM report. While two of our three vegetarian or vegan models contained seafood and a lacto-vegetarian diet can contain seaweed, we were unable to model iodine adequacy in these patterns as iodine is not included in Food Data Central or in the FPM report (16, 25). The development of an iodine database is currently in progress (26).

## Limitations

While this study gives insight into how the HVDP can be adapted for vegetarian eating patterns besides a lacto-ovo vegetarian diet, the models developed in this study are based on idealized versions of the described eating patterns. Modeling can provide an estimate of nutrient adequacy with certain patterns but cannot reflect individual dietary differences or the appropriateness of these patterns for different people. While our models determined overall nutrient adequacy, we are unable to compare our findings to dietary patterns of larger groups of people following vegetarian dietary patterns. Another limitation to this paper and the nature of food pattern modeling is the inability to assess nutrient bioavailability.

## Conclusion

Our models provided insight into the nutritional adequacy of various vegetarian adaptations to the HVDP including lacto-vegetarian, pescatarian, and pescavegan, for generally healthy non-pregnant, non-lactating adults. These models provided adequate amounts of macronutrients and several micronutrients, including some nutrients of concern in vegetarian dietary patterns. The addition of fish (pescatarian model) to the original HVDP increased the amounts of omega-3 fatty acids, vitamin D, and protein. The pescavegan models were the most nutritionally adequate of the three adaptations developed, as they provided adequate amounts of all micronutrients besides vitamin E, vitamin D, and choline. Of the three models, the lacto-vegetarian models provided the least amount of nutrients that are often found in animal products (meat, eggs, fish), such as choline, zinc, and omega-3 fatty acids. This study provides insight into overall nutrient adequacy of various iterations of vegetarian dietary patterns adapted from the DGA.

## Data availability statement

The original contributions presented in this study are included in the article/Supplementary material, further inquiries can be directed to the corresponding author.

## Author contributions

MEC and JMH contributed to the development, organization, writing, and editing of this manuscript. JMH composed the outline. All authors had full access to data and revised and approved the manuscript for publication.
