# Supplementary material for: Modeling lacto-vegetarian, pescatarian, and “pescavegan” USDA food patterns and assessing nutrient adequacy for healthy non-pregnant, non-lactating adults
Source: Front Nutr. 2023 Feb 7;10:1113792. doi: 10.3389/fnut.2023.1113792 (PMC9941140; doi:10.3389/fnut.2023.1113792)
Supplement: Supplementary file 1 [file Data_Sheet_1.docx]

## SUPPLEMENTARY TABLES

## Supplementary Table 1. Seafood food group (1 oz-eq) developed from the 2020 Dietary Guidelines for Americans Food Pattern Modeling Report

| **Nutrients** | **Seafood Food Group** |
| --- | --- |
| **MACRONUTRIENTS** | |
| Calories (kcal) | 39.76 |
| Protein (g) | 6.43 |
| Carbohydrate (g) | 0.12 |
| Fiber (g) | 0.00 |
| Total fat (g) | 1.34 |
| Saturated Fat (g) | 0.30 |
| Monounsaturated fat (g) | 0.44 |
| Polyunsaturated fat (g) | 0.44 |
| Linoleic acid (18:2n-6) (g) | 0.09 |
| Linolenic acid (18:3) (g) | 0.02 |
| EPA (20:5n-3) (g) | 0.06 |
| DHA (22:6n-3) (g) | 0.14 |
| Cholesterol (mg) | 22.85 |
| **MINERALS** | |
| Calcium (mg) | 9.41 |
| Iron (mg) | 0.21 |
| Magnesium (mg) | 9.09 |
| Phosphorus (mg) | 73.85 |
| Potassium (mg) | 91.48 |
| Sodium (mg) | 74.49 |
| Zinc (mg) | 0.33 |
| Copper (mg) | 0.05 |
| Selenium (mg) | 13.87 |
| **VITAMINS** | |
| Vitamin A, RAE (mcg) | 10.51 |
| Vitamin E, AT (mg) | 0.28 |
| Vitamin D (IU) | 69.82 |
| Vitamin C (mg) | 0.40 |
| Thiamin (mg) | 0.03 |
| Riboflavin (mg) | 0.03 |
| Niacin (mg) | 1.55 |
| Vitamin B6 (mg) | 0.09 |
| Vitamin B12 (mcg) | 1.05 |
| Choline (mg) | 19.49 |
| Vitamin K (mcg) | 0.10 |
| Folate (mcg) | 5.09 |

## Supplementary Table 2. Comparison between the dairy food group from the 2020 Dietary Guidelines for Americans Food Pattern Modeling Report and the dairyALT food group^1,2^

|  | **Dairy Food Group** | **DairyALT food group^3^**  Soy milk (11320000)  Soy yogurt (175227) |
| --- | --- | --- |
| **MACRONUTRIENTS** | | |
| Calories (kcal) | 84.84 | 109.48 |
| Protein (g) | 9.05 | 6.34 |
| Carbohydrate (g) | 10.03 | 11.26 |
| Fiber, total dietary (g) | 0.23 | 0.53 |
| Total lipid (fat) (g) | 0.98 | 3.64 |
| Saturated fatty acids (g) | 0.58 | 0.50 |
| Monounsaturated fatty acids (g) | 0.26 | 0.864 |
| Polyunsaturated fatty acids (g) | 0.06 | 1.92^4^ |
| Linoleic acid (18:2n-6) (g) | 0.04 | 1.30^4^ |
| Linolenic acid (18:3) (g) | 0.01 | 0.17^4^ |
| EPA (20:5n-3) (g) | 0.00 | n/a |
| DHA (22:6n-3) (g) | 0.00 | n/a |
| Cholesterol (mg) | 7.39 | 0.00 |
| **MINERALS** | | |
| Calcium (mg) | 306.98 | 301.59 |
| Iron (mg) | 0.08 | 0.94^4^ |
| Magnesium (mg) | 24.82 | 33.6^4^ |
| Phosphorus (mg) | 220.60 | 96.41^4^ |
| Potassium (mg) | 256.54 | 273.61^4^ |
| Sodium (mg) | 193.70 | 108.16 |
| Zinc (mg) | 1.23 | 0.58^4^ |
| Copper (mg) | 0.05 | 0.37^4^ |
| Selenium (mcg) | 7.06 | 5.15^4^ |
| **VITAMINS** | | |
| Vitamin A, RAE (mcg) | 102.80 | 123.03 |
| Vitamin E, AT (mg) | 0.04 | 0.25^4^ |
| Vitamin D, (IU) | 58.80 | 108.80 |
| Vitamin C (mg) | 0.08 | 2.61 |
| Thiamin (mg) | 0.08 | 0.07^4^ |
| Riboflavin (mg) | 0.31 | 0.41^4^ |
| Niacin (mg) | 0.39 | 0.95^4^ |
| Vitamin B6 (mg) | 0.09 | 0.07^4^ |
| Vitamin B12 (mcg) | 0.92 | 1.90^4^ |
| Folate, DFE (mcg) | 9.75 | 20.19^4^ |
| Choline (mg) | 25.67 | 52.89^4^ |
| Vitamin K (mcg) | 0.25 | 6.72^4^ |
| Table originally published in (5) | | |
| 2 Values are per cup equivalent. 1 cup-equivalent = 2.36 dL. AT, alpha=tocopherols; DairyALT, dairy alternative; DFE, dietary folate equivalents; IU, international units; n/a, not available; RAE, retinol activity equivalents | | |
| 3 Numbers denote food codes from the USDA FoodData Central database (27) | | |
| 4 These amounts in the dairyALT food group only reflect data from soy milk. Data on these nutrients were not available in FoodData Central for soy yogurt. | | |

## Supplementary Table 3. Impact of replacing eggs in the 1800 kcal/d Healthy Vegetarian Dietary Pattern (HVDP) with vegetarian protein group foods on Dietary Reference Intakes (DRIs) for females 31-50 y

| **Nutrients** | **DRIs: females 31-50 y** | **HVDP** | **Model 1: Lacto-vegetarian** | **Change from HVDP, %** | **Percentage (%) of DRI met by Model 1** |
| --- | --- | --- | --- | --- | --- |
| **MACRONUTRIENTS** | | | | | |
| Calories kcal | 1800 | 1797 | 1790 | -0.41 | 99.42 |
| Protein (g) | 46 | 76 | 75 | -0.21 | 163.91 |
| Carbohydrate (g) | 130 | 237 | 238 | 0.64 | 183.38 |
| Fiber (g) | 25 | 29 | 29 | 2.19 | 116.80 |
| Total fat (g) | 20-35% | 50 | 49 | -2.07 | Within range |
| Saturated fat (g) | <10% | 10 | 9 | -5.15 | Within limits |
| Monounsaturated fat (g) | n/a | 18 | 18 | -1.53 | n/a |
| Polyunsaturated fat (g) | n/a | 19 | 19 | 0.30 | n/a |
| Linoleic acid (g) | 12 | 17 | 17 | 0.40 | 138.33 |
| Linolenic acid (g) | 1.1 | 2.1 | 2.1 | -0.77 | 190.91 |
| EPA (g) | n/a | 0.000 | 0.000 | 0.00 | n/a |
| DHA (g) | n/a | 0.009 | 0.000 | -100.00 | n/a |
| Cholesterol (mg) | as low as possible | 105 | 25 | -76.23 | n/a |
| **MINERALS** | | | | | |
| Calcium (mg) | 1000 | 1317 | 1318 | 0.06 | 131.75 |
| Iron (mg) | 18 | 16 | 16 | 1.20 | 87.78 |
| Magnesium (mg) | 320 | 365 | 371 | 1.62 | 116.03 |
| Phosphorus (mg) | 700 | 1557 | 1551 | -0.36 | 221.60 |
| Potassium (mg) | 2600 | 3102 | 3113 | 0.34 | 119.73 |
| Sodium (mg) | 2300 | 1421 | 1414 | -0.53 | 61.47 |
| Zinc (mg) | 8 | 11 | 11 | -0.44 | 138.75 |
| Copper (mg) | 0.9 | 2.0 | 1.6 | -20.00 | 177.78 |
| Selenium (mcg) | 55 | 79 | 72 | -7.80 | 131.64 |
| **VITAMINS** | | | | | |
| Vitamin A, RAE (mcg) | 700 | 839 | 807 | -3.82 | 115.24 |
| Vitamin E, AT (mg) | 15 | 9 | 10 | 1.10 | 63.33 |
| Vitamin D (IU) | 600 | 219 | 201 | -8.33 | 33.50 |
| Vitamin C (mg) | 75 | 114 | 115 | 0.05 | 152.67 |
| Thiamin (mg) | 1.1 | 1.7 | 1.8 | 3.09 | 163.64 |
| Riboflavin (mg) | 1.1 | 1.8 | 1.7 | -4.53 | 154.55 |
| Niacin (mg) | 14 | 16 | 16 | 1.20 | 115.71 |
| Vitamin B6 (mg) | 1.3 | 1.7 | 1.7 | -2.20 | 130.77 |
| Vitamin B12 (mcg) | 2.4 | 3.9 | 3.6 | -6.51 | 150.00 |
| Choline (mg) | 425 | 286 | 229 | -19.84 | 53.98 |
| Vitamin K (mcg) | 90 | 133 | 133 | 0.19 | 147.89 |
| Folate, DFE (mcg) | 400 | 594 | 598 | 0.64 | 149.45 |

**Supplementary Table 4. 1800 kcal Pescatarian Model of the Healthy Vegetarian Dietary Pattern (HVDP) compared to Dietary Reference Intakes (DRIs) for females 31-50 y**

| **Nutrients** | **DRIs: females 31-50 y** | **HVDP** | **Model 2: Pescatarian** | **Change from HVDP, %** | **Percentage (%) of DRI met by Model 2** |
| --- | --- | --- | --- | --- | --- |
| **MACRONUTRIENTS** | | | | | |
| Calories kcal | 1800 | 1797 | 1799 | 0.09 | 99.93 |
| Protein (g) | 46 | 76 | 82 | 8.13 | 177.61 |
| Carbohydrate (g) | 130 | 237 | 229 | -3.37 | 176.08 |
| Fiber (g) | 25 | 28 | 28 | -1.31 | 112.80 |
| Total fat (g) | 20-35% | 50 | 51 | 1.74 | Within range |
| Saturated fat (g) | <10% | 10 | 10 | 2.15 | Within limits |
| Monounsaturated fat (g) | n/a | 18 | 18 | 1.83 | n/a |
| Polyunsaturated fat (g) | n/a | 19 | 19 | 1.90 | n/a |
| Linoleic acid (g) | 12 | 17 | 17 | -0.20 | 137.50 |
| Linolenic acid (g) | 1.1 | 2.1 | 2.1 | -0.77 | 190.91 |
| EPA (g) | n/a | 0.000 | 0.073 | 0.00 | n/a |
| DHA (g) | n/a | 0.009 | 0.165 | 1818.60 | n/a |
| Cholesterol (mg) | as low as possible | 105 | 131 | 24.54 | n/a |
| **MINERALS** | | | | | |
| Calcium | 1000 | 1317 | 1316 | -0.08 | 131.57 |
| Iron (mg) | 18 | 16 | 15 | -1.37 | 85.56 |
| Magnesium (mg) | 320 | 365 | 372 | 1.76 | 116.19 |
| Phosphorus (mg) | 700 | 1557 | 1622 | 4.17 | 231.66 |
| Potassium (mg) | 2600 | 3102 | 3190 | 2.83 | 122.70 |
| Sodium (mg) | 2300 | 1421 | 1454 | 2.30 | 63.21 |
| Zinc (mg) | 8 | 11 | 11 | 2.25 | 142.50 |
| Copper (mg) | 0.9 | 2.0 | 1.5 | -25.00 | 166.67 |
| Selenium (mcg) | 55 | 79 | 91 | 16.14 | 165.82 |
| **VITAMINS** | | | | | |
| Vitamin A, RAE (mcg) | 700 | 839 | 847 | 0.94 | 120.94 |
| Vitamin E, AT (mg) | 15 | 9 | 10 | 3.23 | 64.67 |
| Vitamin D (IU) | 600 | 219 | 298 | 35.82 | 49.63 |
| Vitamin C (mg) | 75 | 114 | 115 | 0.14 | 152.80 |
| Thiamin (mg) | 1.1 | 1.7 | 1.7 | -2.63 | 154.55 |
| Riboflavin (mg) | 1.1 | 1.8 | 1.8 | 1.08 | 163.64 |
| Niacin (mg) | 14 | 16 | 17 | 7.44 | 122.86 |
| Vitamin B6 (mg) | 1.3 | 1.7 | 1.8 | 3.56 | 138.46 |
| Vitamin B12 (mcg) | 2.4 | 3.9 | 5.0 | 29.84 | 208.33 |
| Choline (mg) | 425 | 286 | 307 | 7.18 | 72.16 |
| Vitamin K (mcg) | 90 | 133 | 133 | -0.11 | 147.44 |
| Folate, DFE (mcg) | 400 | 594 | 577 | -2.83 | 144.30 |

## Supplementary Table 5. Impact of adding fish to the 1800 kcal/d vegan adaptation of the Healthy Vegetarian Dietary Pattern (HVDP) on Dietary Reference Intakes (DRIs) for females 31-50 y

| **Nutrients** | **DRIs: females 31-50 y** | **HVDP** | **Model 3: Pescavegan** | **Change from HVDP, %** | **Percentage (%) of DRI met by Model 3** |
| --- | --- | --- | --- | --- | --- |
| **MACRONUTRIENTS** | | | | | |
| Calories kcal | 1800 | 1797 | 1866 | 3.82 | 103.64 |
| Protein (g) | 46 | 76 | 73 | -2.86 | 159.57 |
| Carbohydrate (g) | 130 | 237 | 234 | -1.13 | 180.15 |
| Fiber (g) | 25 | 28 | 30 | 3.94 | 118.80 |
| Total fat (g) | 20-35% | 50 | 58 | 15.76 | Within range |
| Saturated fat (g) | <10% | 10 | 9 | -6.19 | Within limits |
| Monounsaturated fat (g) | n/a | 18 | 20 | 10.22 | n/a |
| Polyunsaturated fat (g) | n/a | 19 | 25 | 31.78 | n/a |
| Linoleic acid (g) | 12 | 17 | 20 | 22.78 | 169.17 |
| Linolenic acid (g) | 1.1 | 2.1 | 2.6 | 22.85 | 236.36 |
| EPA (g) | n/a | 0.000 | 0.073 | n/a | n/a |
| DHA (g) | n/a | 0.009 | 0.156 | 1713.95 | n/a |
| Cholesterol (mg) | as low as possible | 105 | 29 | -72.71 | n/a |
| **MINERALS** | | | | | |
| Calcium | 1000 | 1317 | 1300 | -1.25 | 130.03 |
| Iron (mg) | 18 | 16 | 18 | 16.57 | 101.11 |
| Magnesium (mg) | 320 | 365 | 404 | 10.63 | 126.31 |
| Phosphorus (mg) | 700 | 1557 | 1244 | -20.12 | 177.64 |
| Potassium (mg) | 2600 | 3102 | 3252 | 4.82 | 125.08 |
| Sodium (mg) | 2300 | 1421 | 1190 | -16.29 | 51.73 |
| Zinc (mg) | 8 | 11 | 9 | -15.69 | 117.50 |
| Copper (mg) | 0.9 | 2.0 | 2.6 | 30.00 | 288.89 |
| Selenium (mcg) | 55 | 79 | 79 | 0.99 | 144.18 |
| **VITAMINS** | | | | | |
| Vitamin A, RAE (mcg) | 700 | 839 | 875 | 4.35 | 125.03 |
| Vitamin E, AT (mg) | 15 | 9 | 10 | 9.61 | 68.67 |
| Vitamin D (IU) | 600 | 219 | 430 | 95.93 | 71.60 |
| Vitamin C (mg) | 75 | 114 | 122 | 6.87 | 163.07 |
| Thiamin (mg) | 1.1 | 1.7 | 1.7 | -2.63 | 154.55 |
| Riboflavin (mg) | 1.1 | 1.8 | 2.0 | 12.32 | 181.82 |
| Niacin (mg) | 14 | 16 | 19 | 19.31 | 136.43 |
| Vitamin B6 (mg) | 1.3 | 1.7 | 1.8 | 3.56 | 138.46 |
| Vitamin B12 (mcg) | 2.4 | 3.9 | 7.7 | 99.96 | 320.83 |
| Choline (mg) | 425 | 286 | 332 | 15.88 | 78.02 |
| Vitamin K (mcg) | 90 | 133 | 152 | 14.64 | 169.22 |
| Folate, DFE (mcg) | 400 | 594 | 612 | 3.06 | 153.05 |

## Supplementary Table 6. Impact of replacing eggs in the 2000 kcal/d Healthy Vegetarian Dietary Pattern (HVDP) with vegetarian protein group foods on Dietary Reference Intakes (DRIs) for males 51+ y

| **Nutrients** | **DRIs: Males 51+** | **HVDP** | **Model 1: Lacto-vegetarian** | **Change from HVDP, %** | **Percentage (%) of DRI met by Model 1** |
| --- | --- | --- | --- | --- | --- |
| **MACRONUTRIENTS** | | | | | |
| Calories kcal | 2000 | 1998 | 1991 | -0.34 | 99.55 |
| Protein (g) | 56 | 80 | 80 | 0.21 | 142.86 |
| Carbohydrate (g) | 130 | 250 | 251 | 0.48 | 193.08 |
| Fiber (g) | 28 | 30 | 31 | 3.55 | 110.71 |
| Total fat (g) | 20-35% | 54 | 53 | -2.46 | Within range |
| Saturated fat (g) | <10% | 10 | 10 | -2.30 | Within limits |
| Monounsaturated fat (g) | n/a | 20 | 19 | -2.60 | n/a |
| Polyunsaturated fat (g) | n/a | 21 | 21 | 1.82 | n/a |
| Linoleic acid (g) | 14 | 18 | 18 | -1.02 | 128.57 |
| Linolenic acid (g) | 1.6 | 2.3 | 2.4 | 2.47 | 150.00 |
| EPA (g) | n/a | 0.000 | 0.000 | n/a | n/a |
| DHA (g) | n/a | 0.009 | 0.000 | n/a | n/a |
| Cholesterol (mg) | n/a | 105 | 25 | -76.24 | n/a |
| **MINERALS** | | | | | |
| Calcium (mg) | 1000 | 1341 | 1342 | 0.09 | 134.20 |
| Iron (mg) | 8 | 16 | 17 | 3.49 | 212.50 |
| Magnesium (mg) | 420 | 381 | 387 | 1.45 | 92.14 |
| Phosphorus (mg) | 700 | 1609 | 1604 | -0.29 | 229.14 |
| Potassium (mg) | 3400 | 3272 | 3282 | 0.31 | 96.53 |
| Sodium (mg) | 2300 | 1461 | 1455 | -0.43 | 63.26 |
| Zinc (mg) | 11 | 11 | 11 | -3.98 | 100.00 |
| Copper (mg) | 0.9 | 1.6 | 1.7 | 4.12 | 188.89 |
| Selenium (mcg) | 55 | 79 | 73 | -7.81 | 132.73 |
| **VITAMINS** | | | | | |
| Vitamin A, RAE (mcg) | 900 | 847 | 815 | -3.75 | 90.56 |
| Vitamin E, AT (mg) | 15 | 10 | 10 | -2.36 | 66.67 |
| Vitamin D (IU) | 600 | 220 | 202 | -8.08 | 33.67 |
| Vitamin C (mg) | 90 | 129 | 130 | 0.40 | 144.44 |
| Thiamin (mg) | 1.2 | 1.8 | 1.8 | 0.64 | 150.00 |
| Riboflavin (mg) | 1.3 | 1.8 | 1.7 | -6.76 | 130.77 |
| Niacin (mg) | 16 | 17 | 17 | 3.01 | 106.25 |
| Vitamin B6 (mg) | 1.7 | 1.8 | 1.8 | -1.76 | 105.88 |
| Vitamin B12 (mcg) | 2.4 | 3.9 | 3.6 | -6.51 | 150.00 |
| Choline (mg) | 550 | 300 | 243 | -18.88 | 44.18 |
| Vitamin K (mcg) | 120 | 139 | 139 | 0.36 | 115.83 |
| Folate, DFE (mcg) | 400 | 612 | 616 | 0.65 | 154.00 |

**Supplementary Table 7. 2000 kcal Pescatarian Model of the Healthy Vegetarian Dietary Pattern (HVDP) compared to Dietary Reference Intakes (DRIs) for Males 51+ y**

| **Nutrients** | **DRIs: Males 51+** | **HVDP** | **Model 2: Pescatarian** | **Change from HVDP, %** | **Percentage (%) of DRI met by Model 2** |
| --- | --- | --- | --- | --- | --- |
| **MACRONUTRIENTS** | | | | | |
| Calories kcal | 2000 | 1998 | 2043 | 2.27 | 102.15 |
| Protein (g) | 56 | 80 | 87 | 8.97 | 155.36 |
| Carbohydrate (g) | 130 | 250 | 250 | 0.08 | 192.31 |
| Fiber (g) | 28 | 30 | 30 | 0.21 | 107.14 |
| Total fat (g) | 20-35% | 54 | 56 | 3.06 | within range |
| Saturated fat (g) | <10% | 10 | 11 | 7.46 | Within limits |
| Monounsaturated fat (g) | n/a | 20 | 20 | 2.53 | n/a |
| Polyunsaturated fat (g) | n/a | 21 | 21 | 1.82 | n/a |
| Linoleic acid (g) | 14 | 18 | 18 | -1.02 | 128.57 |
| Linolenic acid (g) | 1.6 | 2.3 | 2.3 | -1.80 | 143.75 |
| EPA (g) | n/a | 0.000 | 0.073 | n/a | n/a |
| DHA (g) | n/a | 0.009 | 0.165 | 1818.60 | n/a |
| Cholesterol (mg) | as low as possible | 105 | 131 | 24.48 | n/a |
| **MINERALS** | | | | | |
| Calcium | 1000 | 1341 | 1351 | 0.76 | 135.10 |
| Iron (mg) | 8 | 16 | 17 | 3.49 | 212.50 |
| Magnesium (mg) | 420 | 381 | 392 | 2.77 | 93.33 |
| Phosphorus (mg) | 700 | 1609 | 1693 | 5.24 | 241.86 |
| Potassium (mg) | 3400 | 3272 | 3376 | 3.19 | 99.29 |
| Sodium (mg) | 2300 | 1461 | 1546 | 5.80 | 67.22 |
| Zinc (mg) | 11 | 11 | 12 | 4.75 | 109.09 |
| Copper (mg) | 0.9 | 1.6 | 1.7 | 4.12 | 188.89 |
| Selenium (mcg) | 55 | 79 | 95 | 19.97 | 172.73 |
| **VITAMINS** | | | | | |
| Vitamin A, RAE (mcg) | 900 | 847 | 859 | 1.44 | 95.44 |
| Vitamin E, AT (mg) | 15 | 10 | 11 | 7.41 | 73.33 |
| Vitamin D (IU) | 600 | 220 | 299 | 36.06 | 49.83 |
| Vitamin C (mg) | 90 | 129 | 130 | 0.40 | 144.44 |
| Thiamin (mg) | 1.2 | 1.8 | 1.7 | -4.95 | 141.67 |
| Riboflavin (mg) | 1.3 | 1.8 | 1.8 | -1.28 | 138.46 |
| Niacin (mg) | 16 | 17 | 18 | 9.07 | 112.50 |
| Vitamin B6 (mg) | 1.7 | 1.8 | 1.9 | 3.70 | 111.76 |
| Vitamin B12 (mcg) | 2.4 | 3.9 | 5.0 | 29.84 | 208.33 |
| Choline (mg) | 550 | 300 | 322 | 7.50 | 58.55 |
| Vitamin K (mcg) | 120 | 139 | 139 | 0.36 | 115.83 |
| Folate, DFE (mcg) | 400 | 612 | 618 | 0.98 | 154.50 |

**Supplementary Table 8. Impact of adding fish to the 2000 kcal/d vegan adaptation of the Healthy Vegetarian Dietary Pattern (HVDP) compared to Dietary Reference Intakes (DRIs) for Males 51+ y**

| **Nutrients** | **DRIs: Males 51+** | **HVDP, 2000 kcal** | **Model 3: Pescavegan** | **Change from HVDP, %** | **Percentage (%) of DRI met by Model 3** |
| --- | --- | --- | --- | --- | --- |
| **MACRONUTRIENTS** | | | | | |
| Calories kcal | 2000 | 1998 | 2067 | 3.47 | 103.35 |
| Protein (g) | 56 | 80 | 78 | -2.30 | 139.29 |
| Carbohydrate (g) | 130 | 250 | 247 | -1.12 | 190.00 |
| Fiber (g) | 28 | 30 | 31 | 3.55 | 110.71 |
| Total fat (g) | 20-35% | 54 | 62 | 14.10 | Within range |
| Saturated fat (g) | <10% | 10 | 10 | -2.30 | Within limits |
| Monounsaturated fat (g) | n/a | 20 | 21 | 7.65 | n/a |
| Polyunsaturated fat (g) | n/a | 21 | 27 | 30.91 | n/a |
| Linoleic acid (g) | 14 | 18 | 22 | 20.97 | 157.14 |
| Linolenic acid (g) | 1.6 | 2.3 | 2.8 | 19.55 | 175.00 |
| EPA (g) | n/a | 0.000 | 0.073 | n/a | n/a |
| DHA (g) | n/a | 0.009 | 0.156 | 1713.95 | n/a |
| Cholesterol (mg) | n/a | 105 | 29 | -72.44 | n/a |
| **MINERALS** | | | | | |
| Calcium | 1000 | 1341 | 1325 | -1.17 | 132.50 |
| Iron (mg) | 8 | 16 | 19 | 15.67 | 237.50 |
| Magnesium (mg) | 420 | 381 | 420 | 10.11 | 100.00 |
| Phosphorus (mg) | 700 | 1609 | 1296 | -19.44 | 185.14 |
| Potassium (mg) | 3400 | 3272 | 3422 | 4.59 | 100.65 |
| Sodium (mg) | 2300 | 1461 | 1231 | -15.76 | 53.52 |
| Zinc (mg) | 11 | 11 | 10 | -12.71 | 90.91 |
| Copper (mg) | 0.9 | 1.6 | 2.7 | 65.37 | 300.00 |
| Selenium (mcg) | 55 | 79 | 80 | 1.03 | 145.45 |
| **VITAMINS** | | | | | |
| Vitamin A, RAE (mcg) | 900 | 847 | 883 | 4.28 | 98.11 |
| Vitamin E, AT (mg) | 15 | 10 | 11 | 7.41 | 73.33 |
| Vitamin D (IU) | 600 | 220 | 430 | 95.68 | 71.67 |
| Vitamin C (mg) | 90 | 129 | 137 | 5.81 | 152.22 |
| Thiamin (mg) | 1.2 | 1.8 | 1.7 | -4.95 | 141.67 |
| Riboflavin (mg) | 1.3 | 1.8 | 2.0 | 9.69 | 153.85 |
| Niacin (mg) | 16 | 17 | 20 | 21.18 | 125.00 |
| Vitamin B6 (mg) | 1.7 | 1.8 | 1.9 | 3.70 | 111.76 |
| Vitamin B12 (mcg) | 2.4 | 3.9 | 7.7 | 99.96 | 320.83 |
| Choline (mg) | 550 | 300 | 345 | 15.17 | 62.73 |
| Vitamin K (mcg) | 120 | 139 | 158 | 14.08 | 131.67 |
| Folate, DFE (mcg) | 400 | 612 | 631 | 3.10 | 157.75 |

**Supplementary Table 9. Impact of replacing eggs in the 2200 kcal/d Healthy Vegetarian Dietary Pattern (HVDP) with vegetarian protein group foods on Dietary Reference Intakes (DRIs) for Males 31-50 y**

| **Nutrients** | **DRIs: Males 31-50** | **HVDP** | **Model 1: Lacto-vegetarian** | **Change from HVDP, %** | **Percentage (%) of DRI met by Model 1** |
| --- | --- | --- | --- | --- | --- |
| **MACRONUTRIENTS** | | | | | |
| Calories kcal | 2200 | 2201 | 2197 | -0.20 | 99.86 |
| Protein (g) | 56 | 85 | 85 | 0.05 | 151.79 |
| Carbohydrate (g) | 130 | 277 | 279 | 0.62 | 214.62 |
| Fiber (g) | 31 | 34 | 35 | 3.42 | 112.90 |
| Total fat (g) | 20-35% | 59 | 58 | -0.94 | Within range |
| Saturated fat (g) | <10% | 11 | 10 | -8.69 | Within limits |
| Monounsaturated fat (g) | n/a | 21 | 21 | 0.05 | n/a |
| Polyunsaturated fat (g) | n/a | 22 | 22 | -1.34 | n/a |
| Linoleic acid (g) | 17 | 20 | 20 | 1.73 | 117.65 |
| Linolenic acid (g) | 1.6 | 2.5 | 2.6 | 2.31 | 162.50 |
| EPA (g) | n/a | 0.000 | 0.000 | n/a | n/a |
| DHA (g) | n/a | 0.009 | 0.000 | -100.00 | n/a |
| Cholesterol (mg) | As low as possible | 106 | 25 | -76.34 | n/a |
| **MINERALS** | | | | | |
| Calcium (mg) | 1000 | 1392 | 1393 | 0.10 | 139.30 |
| Iron (mg) | 8 | 18 | 19 | 3.09 | 237.50 |
| Magnesium (mg) | 420 | 419 | 426 | 1.64 | 101.43 |
| Phosphorus (mg) | 700 | 1718 | 1716 | -0.11 | 245.14 |
| Potassium (mg) | 3400 | 3575 | 3595 | 0.56 | 105.74 |
| Sodium (mg) | 2300 | 1573 | 1567 | -0.39 | 68.13 |
| Zinc (mg) | 11 | 12 | 12 | -3.35 | 109.09 |
| Copper (mg) | 0.9 | 1.8 | 1.8 | 0.87 | 200.00 |
| Selenium (mcg) | 55 | 87 | 81 | -6.60 | 147.27 |
| **VITAMINS** | | | | | |
| Vitamin A, RAE (mcg) | 900 | 916 | 884 | -3.51 | 98.22 |
| Vitamin E, AT (mg) | 15 | 11 | 11 | -1.02 | 73.33 |
| Vitamin D (IU) | 600 | 223 | 205 | -8.05 | 34.17 |
| Vitamin C (mg) | 90 | 142 | 142 | 0.09 | 157.78 |
| Thiamin (mg) | 1.2 | 2.0 | 2.0 | -0.62 | 166.67 |
| Riboflavin (mg) | 1.3 | 1.9 | 1.8 | -6.54 | 138.46 |
| Niacin (mg) | 16 | 19 | 19 | 2.33 | 118.75 |
| Vitamin B6 (mg) | 1.3 | 2.0 | 2.0 | -1.39 | 153.85 |
| Vitamin B12 (mcg) | 2.4 | 4.0 | 3.7 | -6.94 | 154.17 |
| Choline (mg) | 550 | 318 | 262 | -17.52 | 47.64 |
| Vitamin K (mcg) | 120 | 170 | 170 | -0.03 | 141.67 |
| Folate, DFE (mcg) | 400 | 697 | 704 | 1.00 | 176.00 |

**Supplementary Table 10. 2200 kcal Pescatarian Model of the Healthy Vegetarian Dietary Pattern (HVDP) compared to Dietary Reference Intakes (DRIs) for Males 31-50 y**

| **Nutrients** | **DRIs: Males 31-50** | **HVDP** | **Model 2: Pescatarian** | **Change from HVDP, %** | **Percentage (%) of DRI met by Model 2** |
| --- | --- | --- | --- | --- | --- |
| **MACRONUTRIENTS** | | | | | |
| Calories kcal | 2200 | 2201 | 2209 | 0.35 | 100.41 |
| Protein (g) | 56 | 85 | 92 | 8.29 | 164.29 |
| Carbohydrate (g) | 130 | 277 | 269 | -2.99 | 206.92 |
| Fiber (g) | 31 | 34 | 33 | -2.49 | 106.45 |
| Total fat (g) | 20-35% | 59 | 60 | 2.48 | Within range |
| Saturated fat (g) | <10% | 11 | 11 | 0.44 | Within limits |
| Monounsaturated fat (g) | n/a | 21 | 21 | 0.05 | n/a |
| Polyunsaturated fat (g) | n/a | 22 | 23 | 3.14 | n/a |
| Linoleic acid (g) | 17 | 20 | 20 | 1.73 | 117.65 |
| Linolenic acid (g) | 1.6 | 2.5 | 2.5 | -1.62 | 156.25 |
| EPA (g) | n/a | 0.000 | 0.082 | n/a | n/a |
| DHA (g) | n/a | 0.009 | 0.185 | 2051.16 | n/a |
| Cholesterol (mg) | as low as possible | 106 | 135 | 27.79 | n/a |
| **MINERALS** | | | | | |
| Calcium | 1000 | 1392 | 1392 | 0.03 | 139.20 |
| Iron (mg) | 8 | 18 | 18 | -2.33 | 225.00 |
| Magnesium (mg) | 420 | 419 | 427 | 1.88 | 101.67 |
| Phosphorus (mg) | 700 | 1718 | 1794 | 4.43 | 256.29 |
| Potassium (mg) | 3400 | 3575 | 3676 | 2.83 | 108.12 |
| Sodium (mg) | 2300 | 1573 | 1617 | 2.79 | 70.30 |
| Zinc (mg) | 11 | 12 | 13 | 4.70 | 118.18 |
| Copper (mg) | 0.9 | 1.8 | 1.8 | 0.87 | 200.00 |
| Selenium (mcg) | 55 | 87 | 101 | 16.46 | 183.64 |
| **VITAMINS** | | | | | |
| Vitamin A, RAE (mcg) | 900 | 916 | 926 | 1.07 | 102.89 |
| Vitamin E, AT (mg) | 15 | 11 | 11 | -1.02 | 73.33 |
| Vitamin D (IU) | 600 | 223 | 312 | 39.94 | 52.00 |
| Vitamin C (mg) | 90 | 142 | 142 | 0.09 | 157.78 |
| Thiamin (mg) | 1.2 | 2.0 | 2.0 | -0.62 | 166.67 |
| Riboflavin (mg) | 1.3 | 1.9 | 1.9 | -1.35 | 146.15 |
| Niacin (mg) | 16 | 19 | 20 | 7.72 | 125.00 |
| Vitamin B6 (mg) | 1.3 | 2.0 | 2.1 | 3.54 | 161.54 |
| Vitamin B12 (mcg) | 2.4 | 4.0 | 5.3 | 33.31 | 220.83 |
| Choline (mg) | 550 | 318 | 341 | 7.35 | 62.00 |
| Vitamin K (mcg) | 120 | 170 | 170 | -0.03 | 141.67 |
| Folate, DFE (mcg) | 400 | 697 | 681 | -2.30 | 170.25 |

**Supplementary Table 11. Impact of adding fish to the 2200 kcal/d vegan adaptation of the Healthy Vegetarian Dietary Pattern (HVDP) on Dietary Reference Intakes (DRIs) for males 31-50 y**

| **Nutrients** | **DRIs: Males 31-50** | **HVDP** | **Model 3: Pescavegan** | **Change from HVDP, %** | **Percentage (%) of DRI met by Model 3** |
| --- | --- | --- | --- | --- | --- |
| **MACRONUTRIENTS** | | | | | |
| Calories kcal | 2200 | 2201 | 2279 | 3.53 | 103.59 |
| Protein (g) | 56 | 85 | 84 | -1.13 | 150.00 |
| Carbohydrate (g) | 130 | 277 | 275 | -0.82 | 211.54 |
| Fiber (g) | 31 | 34 | 35 | 3.42 | 112.90 |
| Total fat (g) | 20-35% | 59 | 67 | 14.43 | Within range |
| Saturated fat (g) | <10% | 11 | 10 | -8.69 | Within limits |
| Monounsaturated fat (g) | n/a | 21 | 23 | 9.58 | n/a |
| Polyunsaturated fat (g) | n/a | 22 | 28 | 25.57 | n/a |
| Linoleic acid (g) | 17 | 20 | 23 | 16.99 | 135.29 |
| Linolenic acid (g) | 1.6 | 2.5 | 3.1 | 21.99 | 193.75 |
| EPA (g) | n/a | 0.000 | 0.082 | n/a | n/a |
| DHA (g) | n/a | 0.009 | 0.177 | 1958.14 | n/a |
| Cholesterol (mg) | As low as possible | 106 | 33 | -68.76 | n/a |
| **MINERALS** | | | | | |
| Calcium | 1000 | 1392 | 1378 | -0.97 | 137.80 |
| Iron (mg) | 8 | 18 | 21 | 13.95 | 262.50 |
| Magnesium (mg) | 420 | 419 | 460 | 9.76 | 109.52 |
| Phosphorus (mg) | 700 | 1718 | 1419 | -17.40 | 202.71 |
| Potassium (mg) | 3400 | 3575 | 3746 | 4.79 | 110.18 |
| Sodium (mg) | 2300 | 1573 | 1354 | -13.93 | 58.87 |
| Zinc (mg) | 11 | 12 | 11 | -11.41 | 100.00 |
| Copper (mg) | 0.9 | 1.8 | 2.8 | 56.91 | 311.11 |
| Selenium (mcg) | 55 | 87 | 90 | 3.78 | 163.64 |
| **VITAMINS** | | | | | |
| Vitamin A, RAE (mcg) | 900 | 916 | 954 | 4.13 | 106.00 |
| Vitamin E, AT (mg) | 15 | 11 | 12 | 7.98 | 80.00 |
| Vitamin D (IU) | 600 | 223 | 444 | 99.15 | 74.00 |
| Vitamin C (mg) | 90 | 142 | 150 | 5.72 | 166.67 |
| Thiamin (mg) | 1.2 | 2.0 | 2.0 | -0.62 | 166.67 |
| Riboflavin (mg) | 1.3 | 1.9 | 2.1 | 9.03 | 161.54 |
| Niacin (mg) | 16 | 19 | 22 | 18.49 | 137.50 |
| Vitamin B6 (mg) | 1.3 | 2.0 | 2.1 | 3.54 | 161.54 |
| Vitamin B12 (mcg) | 2.4 | 4.0 | 8.0 | 101.22 | 333.33 |
| Choline (mg) | 550 | 318 | 367 | 15.53 | 66.73 |
| Vitamin K (mcg) | 120 | 170 | 190 | 11.73 | 158.33 |
| Folate, DFE (mcg) | 400 | 697 | 719 | 3.16 | 179.75 |

**Supplementary Table 12. Impact of replacing eggs in the 2400 kcal/d Healthy Vegetarian Dietary Pattern (HVDP) with vegetarian protein group foods on Dietary Reference Intakes (DRIs) for males ages 19-30 y**

| **Nutrients** | **DRIs: Males 19-30** | **HVDP** | **Model 1: Lacto-vegetarian** | **Change from HVDP, %** | **Percentage (%) of DRI met by Model 1** |
| --- | --- | --- | --- | --- | --- |
| **MACRONUTRIENTS** | | | | | |
| Calories kcal | 2400 | 2404 | 2400 | -0.18 | 100.00 |
| Protein (g) | 56 | 91 | 91 | -0.11 | 162.50 |
| Carbohydrate (g) | 130 | 297 | 299 | 0.58 | 230.00 |
| Fiber (g) | 34 | 37 | 37 | 1.19 | 108.82 |
| Total fat (g) | 20-35% | 63 | 62 | -1.97 | Within range |
| Saturated fat (g) | <10% | 12 | 11 | -6.32 | Within limit |
| Monounsaturated fat (g) | n/a | 23 | 22 | -2.99 | n/a |
| Polyunsaturated fat (g) | n/a | 24 | 24 | -0.75 | n/a |
| Linoleic acid (g) | 17 | 21 | 21 | -1.61 | 123.53 |
| Linolenic acid (g) | 1.6 | 2.7 | 2.8 | 2.29 | 175.00 |
| EPA (g) | n/a | 0.000 | 0.000 | n/a | n/a |
| DHA (g) | n/a | 0.009 | 0.000 | -100.00 | n/a |
| Cholesterol (mg) | n/a | 106 | 26 | -75.48 | n/a |
| **MINERALS** | | | | | |
| Calcium (mg) | 1000 | 1436 | 1437 | 0.07 | 143.70 |
| Iron (mg) | 8 | 20 | 21 | 3.20 | 262.50 |
| Magnesium (mg) | 400 | 450 | 456 | 1.44 | 114.00 |
| Phosphorus (mg) | 700 | 1822 | 1819 | -0.18 | 259.86 |
| Potassium (mg) | 3400 | 3704 | 3719 | 0.41 | 109.38 |
| Sodium (mg) | 2300 | 1684 | 1676 | -0.46 | 72.87 |
| Zinc (mg) | 11 | 13 | 13 | -2.52 | 118.18 |
| Copper (mg) | 0.9 | 1.9 | 2.0 | 3.77 | 222.22 |
| Selenium (mcg) | 55 | 94 | 88 | -6.45 | 160.00 |
| **VITAMINS** | | | | | |
| Vitamin A, RAE (mcg) | 900 | 935 | 901 | -3.69 | 100.11 |
| Vitamin E, AT (mg) | 15 | 12 | 12 | 0.49 | 80.00 |
| Vitamin D (IU) | 600 | 227 | 208 | -8.20 | 34.67 |
| Vitamin C (mg) | 90 | 143 | 142 | -0.39 | 157.78 |
| Thiamin (mg) | 1.2 | 2.2 | 2.2 | 0.69 | 183.33 |
| Riboflavin (mg) | 1.3 | 2.0 | 1.9 | -5.18 | 146.15 |
| Niacin (mg) | 16 | 20 | 20 | -1.08 | 125.00 |
| Vitamin B6 (mg) | 1.3 | 2.1 | 2.1 | -1.40 | 161.54 |
| Vitamin B12 (mcg) | 2.4 | 4.1 | 3.9 | -4.90 | 162.50 |
| Choline (mg) | 550 | 332 | 275 | -17.12 | 50.00 |
| Vitamin K (mcg) | 120 | 174 | 172 | -1.15 | 143.33 |
| Folate, DFE (mcg) | 400 | 766 | 772 | 0.78 | 193.00 |

**Supplementary Table 13. 2400 kcal Pescatarian Model of the Healthy Vegetarian Dietary Pattern (HVDP) compared to Dietary Reference Intakes (DRIs) for Males 19-30 y**

| **Nutrients** | **DRIs: Males 19-30** | **HVDP** | **Model 2: Pescatarian** | **Change from HVDP, %** | **Percentage (%) of DRI met by Model 2** |
| --- | --- | --- | --- | --- | --- |
| **MACRONUTRIENTS** | | | | | |
| Calories kcal | 2400 | 2404 | 2422 | 0.74 | 100.92 |
| Protein (g) | 56 | 91 | 99 | 8.68 | 176.79 |
| Carbohydrate (g) | 130 | 297 | 289 | -2.78 | 222.31 |
| Fiber (g) | 34 | 37 | 36 | -1.54 | 105.88 |
| Total fat (g) | 20-35% | 63 | 65 | 2.77 | Within range |
| Saturated fat (g) | <10% | 12 | 12 | 2.19 | Within limits |
| Monounsaturated fat (g) | n/a | 23 | 23 | 1.42 | n/a |
| Polyunsaturated fat (g) | n/a | 24 | 25 | 3.39 | n/a |
| Linoleic acid (g) | 17 | 21 | 21 | -1.61 | 123.53 |
| Linolenic acid (g) | 1.6 | 2.7 | 2.7 | -1.36 | 168.75 |
| EPA (g) | n/a | 0.000 | 0.091 | n/a | n/a |
| DHA (g) | n/a | 0.009 | 0.205 | 2283.72 | n/a |
| Cholesterol (mg) | n/a | 106 | 139 | 31.06 | n/a |
| **MINERALS** | | | | | |
| Calcium | 1000 | 1436 | 1437 | 0.07 | 143.70 |
| Iron (mg) | 8 | 20 | 20 | -1.71 | 250.00 |
| Magnesium (mg) | 400 | 450 | 458 | 1.89 | 114.50 |
| Phosphorus (mg) | 700 | 1822 | 1908 | 4.70 | 272.57 |
| Potassium (mg) | 3400 | 3704 | 3814 | 2.98 | 112.18 |
| Sodium (mg) | 2300 | 1684 | 1738 | 3.22 | 75.57 |
| Zinc (mg) | 11 | 13 | 14 | 4.98 | 127.27 |
| Copper (mg) | 0.9 | 1.9 | 2.0 | 3.77 | 222.22 |
| Selenium (mcg) | 55 | 94 | 111 | 18.00 | 201.82 |
| **VITAMINS** | | | | | |
| Vitamin A, RAE (mcg) | 900 | 935 | 944 | 0.91 | 104.89 |
| Vitamin E, AT (mg) | 15 | 12 | 12 | 0.49 | 80.00 |
| Vitamin D (IU) | 600 | 227 | 325 | 43.43 | 54.17 |
| Vitamin C (mg) | 90 | 143 | 142 | -0.39 | 157.78 |
| Thiamin (mg) | 1.2 | 2.2 | 2.1 | -3.89 | 175.00 |
| Riboflavin (mg) | 1.3 | 2.0 | 2.0 | -0.18 | 153.85 |
| Niacin (mg) | 16 | 20 | 22 | 8.82 | 137.50 |
| Vitamin B6 (mg) | 1.3 | 2.1 | 2.2 | 3.29 | 169.23 |
| Vitamin B12 (mcg) | 2.4 | 4.1 | 5.6 | 36.56 | 233.33 |
| Choline (mg) | 550 | 332 | 358 | 7.89 | 65.09 |
| Vitamin K (mcg) | 120 | 174 | 171 | -1.73 | 142.50 |
| Folate, DFE (mcg) | 400 | 766 | 750 | -2.09 | 187.50 |

**Supplementary Table 14. Impact of adding fish to the 2400 kcal/d vegan adaptation of the Healthy Vegetarian Dietary Pattern (HVDP) on Dietary Reference Intakes (DRIs) for males 19-30**

| **Nutrients** | **DRIs: Males 19-30** | **HVDP** | **Model 3: Pescavegan** | **Change from HVDP, %** | **Percentage (%) of DRI met by Model 3** |
| --- | --- | --- | --- | --- | --- |
| **MACRONUTRIENTS** | | | | | |
| Calories kcal | 2400 | 2404 | 2488 | 3.48 | 103.67 |
| Protein (g) | 56 | 91 | 91 | -0.11 | 162.50 |
| Carbohydrate (g) | 130 | 297 | 295 | -0.76 | 226.92 |
| Fiber (g) | 34 | 37 | 38 | 3.93 | 111.76 |
| Total fat (g) | 20-35% | 63 | 72 | 13.84 | Within range |
| Saturated fat (g) | <10% | 12 | 11 | -6.32 | Within limit |
| Monounsaturated fat (g) | n/a | 23 | 25 | 10.24 | n/a |
| Polyunsaturated fat (g) | n/a | 24 | 30 | 24.06 | n/a |
| Linoleic acid (g) | 17 | 21 | 25 | 17.14 | 147.06 |
| Linolenic acid (g) | 1.6 | 2.7 | 3.3 | 20.56 | 206.25 |
| EPA (g) | n/a | 0.000 | 0.091 | n/a | n/a |
| DHA (g) | n/a | 0.009 | 0.196 | 2179.07 | n/a |
| Cholesterol (mg) | n/a | 106 | 36 | -66.06 | n/a |
| **MINERALS** | | | | | |
| Calcium | 100 | 1436 | 1422 | -0.97 | 142.20 |
| Iron (mg) | 8 | 20 | 23 | 13.03 | 287.50 |
| Magnesium (mg) | 400 | 450 | 492 | 9.45 | 123.00 |
| Phosphorus (mg) | 700 | 1822 | 1533 | -15.87 | 219.00 |
| Potassium (mg) | 3400 | 3704 | 3884 | 4.87 | 114.24 |
| Sodium (mg) | 2300 | 1684 | 1474 | -12.46 | 64.09 |
| Zinc (mg) | 11 | 13 | 12 | -10.02 | 109.09 |
| Copper (mg) | 0.9 | 1.9 | 3.0 | 55.66 | 333.33 |
| Selenium (mcg) | 55 | 94 | 99 | 5.24 | 180.00 |
| **VITAMINS** | | | | | |
| Vitamin A, RAE (mcg) | 900 | 935 | 972 | 3.90 | 108.00 |
| Vitamin E, AT (mg) | 15 | 12 | 13 | 8.86 | 86.67 |
| Vitamin D (IU) | 600 | 227 | 457 | 101.69 | 76.17 |
| Vitamin C (mg) | 90 | 143 | 150 | 5.22 | 166.67 |
| Thiamin (mg) | 1.2 | 2.2 | 2.1 | -3.89 | 175.00 |
| Riboflavin (mg) | 1.3 | 2.0 | 2.2 | 9.80 | 169.23 |
| Niacin (mg) | 16 | 20 | 24 | 18.71 | 150.00 |
| Vitamin B6 (mg) | 1.3 | 2.1 | 2.2 | 3.29 | 169.23 |
| Vitamin B12 (mcg) | 2.4 | 4.1 | 8.3 | 102.40 | 345.83 |
| Choline (mg) | 550 | 332 | 383 | 15.43 | 69.64 |
| Vitamin K (mcg) | 120 | 174 | 191 | 9.76 | 159.17 |
| Folate, DFE (mcg) | 400 | 766 | 788 | 2.87 | 197.00 |
